# Motion analysis of the wrist joints in Chinese rheumatoid arthritis patients: a cross-sectional study

**DOI:** 10.1186/s12891-018-2146-z

**Published:** 2018-07-28

**Authors:** Lijuan Zhang, Haixia Cao, Qiuxiang Zhang, Ting Fu, Rulan Yin, Yunfei Xia, Liren Li, Zhifeng Gu

**Affiliations:** 1 Department of Rheumatology, Affiliated Hospital of Nantong University, 20th Xisi Road, 226001 Nantong, People’s Republic of China; 2 Department of Rheumatology, Ruijin Hospital, Shanghai Jiao Tong University School of Medicine, 197 Ruijin 2nd Road, Shanghai, 200025 China; 3 School of Nursing, Nantong University, 19th Qixiu Road, 226001 Nantong, People’s Republic of China

## Abstract

**Background:**

The wrist is often severely affected in rheumatoid arthritis (RA) patients; however, little is known about the potential risk factors of the reduced wrist range of motion. In this study, we explored a broad range of possible risk factors of wrist range of motion in RA patients. We also determined whether measurements of wrist range of motion reflect Sharp score for the wrists.

**Methods:**

Active wrist volar flexion, dorsal flexion, radial deviation and ulnar deviation were assessed using a goniometer. RA patients underwent standardized laboratory and radiographic examinations and completed several questionnaires. A linear regression model was used to study association between the wrist range of motion and independent variables. In addition, Spearman and Pearson correlation analysis were used to compare influence factors and outcome measurements between the measurements of wrist range of motion and Sharp score for the wrists.

**Results:**

In this study, lower socioeconomic status, longer disease duration, severe pain, higher disease activity and drug treatments were associated with reduced wrist range of motion in RA patients (*n* = 102, 86.3% female, mean ± SD age, 55.0 ± 11.7 years, and mean ± SD disease duration, 8.4 ± 8.7 years). Furthermore, wrist range of motion was highly correlated with Sharp score for the wrists (*P* < 0.05).

**Conclusions:**

Socioeconomic status and disease-specific factors were significantly associated with wrist range of motion in RA patients. The results indicated that rheumatologists and nurses should note the measurements of wrist range of motion in RA patients, especially those with a low socioeconomic status, a long disease duration, severe pain, and high disease activity to develop strategies to improve their quality of life.

## Background

Rheumatoid arthritis (RA) is a chronic, inflammatory, progressive autoimmune disease that causes pain, limited range of motion (ROM) of joints, and joints destruction [1], and seriously impacts patients’ psychological [2] and physical [3] well-being. The wrist was affected in 50% of patients with RA during the first 2 years after onset of the disease, increasing to more than 90% after 10 years [4]. There was increasing evidence that reduced wrist ROM was associated with RA patients’ functional disability [1, 5]. Furthermore, evaluation of wrist ROM was important in the therapeutic approach to patients with RA [6], and increasing joints motion was a particular goal of the surgical treatments for rheumatic wrist joints [5]. Therefore, it is important to examine which factors have influence on ROM, especially in the wrist.

Several studies have suggested that ROM was associated with age, gender [7], disease duration [8], pain [9, 10], disease activity [10], medical therapy [11, 12], laboratory indexes [13], and disability [1, 14]. It has been reported that RA patients had a higher prevalence of anxiety and depression compared with the general population [2], and the disease exerted an unfavorable impact on the quality of life [3]. Our group has reported that socioeconomic status (SES) was significantly associated with patients’ anxiety/depression and quality of life in rheumatic diseases [15, 16], but exact figures about the associations among SES, anxiety/depression, quality of life, and wrist ROM in RA patients were scarce. To minimize activity limitations and maintain quality of life, it is important for health professionals to increase RA patients’ ROM and provide effective treatments.

To date, Sharp score has assumed a paramount position in the evaluation of RA patients with joint damage in hand-wrist joints. Previous studies have reported that female [17], age [18, 19], body mass index (BMI) [20–22], socioeconomic status (SES) [23], disease duration [20, 24, 25], disease activity [26, 27], comorbid conditions [28, 29], erythrocyte sedimentation rate (ESR) [17], and rheumatoid factor (RF) [24, 30–32] were associated with joint destruction. However, little is known about the associations between Sharp score and the wrist ROM. Only a study from the USA reported that the number of deformed joints, which was rated on each of 48 joints as normal or abnormal in terms of alignment and ROM, was highly correlated with the total Sharp score in RA patients [33]. In the present study, the relationships between influence factors or outcomes values and Sharp score or ROM measurements for the wrists were analyzed.

Therefore, the aims of the present study were the following: (1) to explore a broad range of possible risk factors of wrist ROM in patients with RA; (2) to determine whether RA patients’ ROM measurements reflect Sharp score for the wrists.

## Methods

### Study participants

Patients who fulfilled the American College of Rheumatology (ACR) criteria (1987 or 2012) for RA were recruited from the Affiliated Hospital of Nantong University from January 2015 to April 2016. Of the RA patients who were consecutively invited to participate in a single-centered cross-sectional study, 102 (91.1% of the patients) took part and completed the relevant questionnaires. Patients were excluded based on either of the following: (1) they were less than 18 years old; (2) they did not complete the questionnaire; (3) they did not complete the measurements of ROM and Sharp score for the wrists. This cross-sectional study was approved by the Ethics Committee of the Affiliated Hospital of Nantong University, and a written informed consent was obtained from each RA patient.

### Primary outcomes

Active ROM was measured bilaterally in the wrist with a goniometer. The goniometer was applied superficially at the dorsum of each respective joint. The angle of wrist volar flexion, dorsal flexion, radial deviation, and ulnar deviation were measured relative to a position of zero degrees. Participants had to carry out the motion with their muscle strength to increase the angle and keep their joints in position. Measurements were carried out by two trained physiotherapists under the supervision of a rheumatologist. Two physiotherapists were trained with procedures among 30 healthy subjects before the trial. They were kept unaware of the measurement data of their counterpart. Measurement procedures were standardized prior to the study. The values used for analysis were the means of the right and left sides.

### Independent variables

At baseline, sociodemographic and disease characteristic [including gender, age (years), BMI (kg/m^2^), disease duration (years), education (years), employment status, income/person/month (Yuan), health insurance, and comorbid conditions] were recorded.

One experienced rheumatologist (GZ) and two rheumatologists (XY and GG) scored joint damage at the same time. Radiographs of both wrists were scored using the van der Heijde-modified Sharp Score (HSS). The total score for the wrists ranged from 0 to 87, with the erosion score (E score) ranging from 0 to 35 and the joint space narrowing score (JSN score) ranging from 0 to 32. All were read by the examiners without the knowledge of the patient identity [34].

As described previously [35], hand grip and pinch strength were measured with a hydraulic hand grip and pinch dynamiter [36]. Physical function was evaluated by the Health Assessment Questionnaire (HAQ) [37]. The Hospital Anxiety and Depression Scale (HADS) was used to assess levels of anxiety and depression [38]. Participants’ health status was assessed using the Short Form 36 (SF-36) [39, 40]. Disease activity was estimated with the valid and reliable 28-joint Disease Activity Score (DAS28) [41–43]. Several serological markers, ESR, C-reactive protein (CRP), and RF measured at the time of diagnosis were examined [44].

The personal medication information was gained by querying the electronic medical records combined with self-reports of patients, including the use of NSAIDs, DMARDs, corticosteroids, and biologics.

We have described questionnaire and measurement administration in detail previously [35]. Briefly, written questionnaires were provided on paper, and all participants completed the questionnaires under a physician’s supervision in a clinical setting.

### Statistical analysis

The data were expressed as the mean ± SD for continuous variables and as frequencies (%) for categorical variables. Descriptive analyses were performed to investigate the participants’ characteristics. The Spearman and Pearson correlations analysis were used to compare the influence factors and outcome measurements between Sharp score and the ROM measurements for the wrists.

Furthermore, variables shown to be significantly associated with the primary outcome in the Spearman and Pearson correlations analysis were included in the multivariable regression analysis to identify the independent factors of wrist ROM. Statistical significance was set at *P* < 0.05 (two-sided). Standardized regression coefficients (*β*) and the *R*-squared (*R*^*2*^) were presented to show the relative importance of the independent variables when compared to each other, and the proportion of the variance in the wrist ROM accounted for by the factors in the multivariable regression model, respectively [45]. Analyses were completed using SPSS version 20.0.

## Results

### Sample characteristics

Ten RA patients did not complete the questionnaires due to lack of interest, resulting in the enrollment of 102 RA patients in the current study. Table 1 presented the baseline participant characteristics included in our analysis. The mean ± SD age of the respondents was 55.0 ± 11.7 years, and 86.3% were female. The mean ± SD disease duration was 8.4 ± 8.7 years, and 93.1 and 35.3% had health insurance and comorbid condition, respectively. The participants tended to have lower income (< 3000 Yuan; 87.2%) and lower than a high school level of education (71.6%). The mean ± SD pain and DAS28 scores of the participants were 43.1 ± 27.2 and 3.7 ± 1.5, respectively. The patients tended to use DMARDs (91.3%), and 78.4% were RF positive. The mean (range) wrist ROM scores, grip/pinch strength, Sharp score, the HAQ score, the HADS anxiety and depression scores, and the scores of SF-36 PCS and MCS were shown in Table 2.

**Table 1:** Baseline characteristics of 102 patients with rheumatoid arthritis

Characteristic/factor	Value
Gender, female, no. (%)	88 (86.3)
Age, mean ± SD years	55.0 ± 11.7
BMI, mean ± SD kg/m2	22.6 ± 3.3
Disease duration, mean ± SD years	8.4 ± 8.7
Education, years, no. (%)	
≤ 9 years	73 (71.6)
> 9 years	29 (28.4)
Employment status, no. (%)	
Full-time work	63 (61.8)
Part-time work	37 (36.2)
Unemployed	2 (2.0)
Income/person/month, Yuan, no. (%)	54 (52.9)
≤ 1000 Yuan	35 (34.3)
1000–3000 Yuan	12 (11.8)
3000–5000 Yuan	1 (1.0)
≥ 5000 Yuan	95 (93.1)
Health insurance, yes, no. (%)	36 (35.3)
Comorbid condition, yes, no. (%)	43.1 ± 27.2
VAS pain (range 0–100), mean ± SD	4.1 ± 6.0
28-TJC, mean ± SD	54 (52.9)
28-SJC, mean ± SD	2.6 ± 3.8
DAS28, mean ± SD	3.7 ± 1.5
NAIDS usage, yes, no. (%)	42 (41.2)
DMARDs usage, yes, no. (%)	93 (91.2)
Corticosteroids usage, yes, no. (%)	43 (42.2)
Biologics usage, yes, no. (%)	4 (3.9)
ESR, mean ± SD mm/h	25.7 ± 24.9
CRP, mean ± SD mg/L	15.5 ± 24.5
RF positivity, yes, no. (%)	80 (78.4)

*BMI* Body mass index, *VAS* Visual analog scale, *TJC* Tender joint count, *SJC* Swollen joint count, *DAS28* Disease activity score in 28 joints, *NSAID* Nonsteroidal anti-inflammatory drugs, *DMARD* Disease modifying anti-rheumatic drugs, *ESR* Erythrocyte sedimentation rate, *CRP* C-reactive protein, *RF* Rheumatoid factor

**Table 2:** Clinical characteristics of 102 patients with rheumatoid arthritis

Characteristic/factor	Value	Range
Wrist volar flexion ǂ, mean ± SD degrees	38.7 ± 18.7	0 to 80
Wrist dorsal flexion ǂ, mean ± SD degrees	35.2 ± 17.2	0 to 65
Wrist ulnar deviation ǂ, mean ± SD degrees	29.7 ± 14.0	0 to 63
Wrist radial deviation ǂ, mean ± SD degrees	13.1 ± 7.8	0 to 35
Sharp score for the wrists, mean ± SD	8.6 ± 6.5	0 to 52
Grip strength ǂ, mean ± SD kg	13.2 ± 8.6	0 to 40
Pinch strength ǂ, mean ± SD kg	3.3 ± 2.2	0 to 11
HAQ score (range 0–3), mean ± SD	0.4 ± 0.6	0 to 2.6
HADS-anxiety score (range 0–21), mean ± SD	9.3 ± 2.7	4 to 17
HADS-depression score (range 0–21), mean ± SD	8.9 ± 2.4	4 to 15
PCS score (range 0–100), mean ± SD	43.9 ± 22.9	2.5 to 92.8
MCS score (range 0–100), mean ± SD	53.0 ± 22.2	1 to 100

ǂ Mean of right and left sides. *HAQ* Health assessment questionnaire, *HADS* Hospital anxiety and depression scale, *PCS* Physical components summary, *MCS* Mental components summary

### Higher sharp score was significantly associated with reduced ROM for the wrists

As shown in Table 2, the mean (range) ROM scores varied from 13.1 (0 to 35) to 38.7 (0 to 80) degrees in the wrist joint actions, which were much lower than the normal reference. This result was in accordance with previous findings [1, 5, 14]. The correlation between the ROM scores of the wrist joint actions ranged from 0.44 to 0.67. This finding confirms the conclusion of Steultjens, et al.*..* that joint ROM cannot be regarded as a unidimensional physical characteristic of osteoarthritis (OA) patients [46]. Furthermore, Orces CH, et al [33] reported that the number of deformed joints was significantly associated with the total Sharp score. We also found that a higher Sharp score was highly correlated with a lower ROM in the wrist (Table 3). Thus, this raised an interesting question of whether RA patients’ ROM measurements might reflect Sharp score for the wrists.

**Table 3:** Correlation between the wrist ROM and Sharp score for the wrists (*N* = 102)

Variable	Wrist volar flexion (degrees)		Wrist dorsal flexion (degrees)		Wrist ulnar deviation (degrees)		Wrist radial deviation (degrees)	
	*r*	*P*	*r*	*P*	*r*	*P*	*r*	*P*
Wrist volar flexion ǂ (degrees)								
Wrist dorsal flexion ǂ (degrees)	0.67**	0.000						
Wrist ulnar deviation ǂ (degrees)	0.62**	0.000	0.64**	0.000				
Wrist radial deviation ǂ (degrees)	0.47**	0.000	0.44**	0.000	0.48**	0.000		
Sharp score for the wrists	− 0.62**	0.000	− 0.63**	0.000	− 0.67**	0.000	− 0.42**	0.000

ǂ Mean of right and left sides. *P < 0.05, **P < 0.01

### SES, disease activity, laboratory indexes and outcome measures were significantly associated with sharp score and ROM for the wrists

As indicated in Table 4, both Sharp score and the ROM for the wrists were correlated to a similar degree with disease duration, employment status, income, comorbid conditions, grip/pinch strength, the HAQ score, the SF-36 PCS and MCS scores (Table 4).

**Table 4:** Correlations among the wrist ROM, Sharp score for the wrists, and the variables used in this study (N = 102)

Variable	Sharp score for the wrists		Wrist volar flexion ǂ (degrees)		Wrist dorsal flexion ǂ (degrees)		Wrist ulnar deviation ǂ (degrees)		Wrist radial deviation ǂ (degrees)	
	*r*	*P*	*r*	*P*	*r*	*P*	*r*	*P*	*r*	*P*
Gender	0.15	0.129	0.02	0.858	0.03	0.746	0.03	0.775	0.18	0.065
Age, years	−0.08	0.451	−0.17	0.096	− 0.08	0.434	− 0.04	0.659	− 0.04	0.713
BMI, kg/m2	−0.06	0.561	−0.03	0.794	−0.12	0.233	−0.08	0.454	−0.08	0.404
Disease duration, years	0.47**	0.000	−0.30**	0.002	−0.46**	0.000	−0.39**	0.000	−0.13	0.200
Education, years	−0.07	0.515	0.26**	0.008	0.20*	0.048	0.21*	0.037	0.16	0.118
Employment status	−0.22*	0.025	0.26*	0.008	0.07	0.458	0.25*	0.012	0.04	0.660
Income/person/month, Yuan	−0.31**	0.002	0.40**	0.000	0.31**	0.002	0.30**	0.002	0.12	0.224
Health insurance	0.05	0.624	0.12	0.248	0.25*	0.012	0.22*	0.027	0.16	0.108
Comorbid condition	0.23*	0.021	−0.18	0.078	−0.27**	0.006	−0.26**	0.007	−0.12	0.215
VAS pain	0.31**	0.002	−0.41**	0.000	−0.40**	0.000	−0.43**	0.000	−0.34**	0.001
28-TJC	0.12	0.229	−0.30**	0.003	−0.30**	0.002	−0.28**	0.005	−0.20*	0.043
28-SJC	0.16	0.102	−0.23*	0.021	−0.25*	0.011	−0.13	0.195	−0.21*	0.038
DAS28	0.27*	0.017	−0.32**	0.001	−0.30**	0.002	−0.31**	0.002	−0.39**	0.000
NAIDS usage	−0.02	0.866	−0.05	0.634	−0.04	0.702	0.00	0.976	0.05	0.627
DMARDs usage	0.00	0.995	0.08	0.438	−0.01	0.958	−0.07	0.477	−0.05	0.604
Corticosteroids usage	0.06	0.529	−0.17	0.080	−0.27**	0.007	−0.11	0.258	−0.06	0.547
Biologics usage	0.04	0.456	0.08	0.438	0.00	0.949	0.05	0.567	0.05	0.447
ESR, mm/h	0.14	0.161	−0.21*	0.039	−0.20*	0.041	−0.24*	0.014	−0.38**	0.000
CPR, mg/L	0.12	0.214	- 0.24*	0.014	−0.29**	0.003	−0.27**	0.006	−0.36**	0.000
RF positivity	0.14	0.155	−0.05	0.619	−0.08	0.448	−0.14	0.163	−0.28**	0.005
Grip strength ǂ (kg)	−0.39**	0.000	0.40**	0.000	0.37**	0.000	0.47**	0.000	0.37**	0.000
Pinch strength ǂ (kg)	−0.30**	0.002	0.29**	0.003	0.25*	0.013	0.32**	0.001	0.24*	0.017
HAQ score	0.35**	0.000	−0.49**	0.000	−0.49**	0.000	−0.47**	0.000	−0.41**	0.000
HADS-anxiety score	0.16	0.109	−0.10	0.322	−0.25*	0.012	−0.06	0.555	−0.24*	0.019
HADS-depression score	0.05	0.610	−0.21*	0.040	−0.20*	0.047	−0.12	0.250	−0.23*	0.020
PCS score	−0.25*	0.011	0.41**	0.000	0.38**	0.000	0.31**	0.002	0.29**	0.003
MCS score	−0.22*	0.023	0.26**	0.008	0.32**	0.001	0.21*	0.030	0.19	0.113

ǂ Mean of right and left sides. *BMI* Body mass index, *VAS* Visual analog scale, *TJC* Tender joint count, *SJC* Swollen joint count, *DAS28* Disease activity score in 28 joints, *NSAID* Nonsteroidal anti-inflammatory drugs, *DMARD* Disease modifying antirheumatic drugs, *ESR* Erythrocyte sedimentation rate, *CRP* C-reactive protein, *RF* Rheumatoid factor, *HAQ* Health assessment, questionnaire, *HADS* Hospital anxiety and depression scale, *PCS* Physical components summary, *MCS* Mental components summary. *P < 0.05, **P < 0.01

### SES and RA disease-specific factors were the potential risk factors of wrist ROM

We used stepwise linear regression analysis to investigate the potential risk factors of wrist ROM, as shown in Table 5. Only the independent variables that were significantly associated with wrist ROM were entered into model. We found that SES and RA disease-specific factors were the important predictors of wrist ROM. In addition, we found that there were significant correlations between corticosteroids usage and lower wrist dorsal flexion, which was in contrast with a previous finding [12]. It might be explained that the radiological and functional damage of the wrist is likely to be a direct by-product of the more severe disease features, while steroid usage is likely to be a consequence of the individual clinical profile with more persistent and/or high disease activity.

**Table 5:** Results of the multivariable analysis of the association between factors and the ROM scores of the different wrist joint actions (degrees) (*N* = 102)

Factor	Wrist volar flexion ǂ (degrees)			Wrist dorsal flexion ǂ (degrees)			Wrist ulnar deviation ǂ (degrees)			Wrist radial deviation ǂ (degrees)		
	*B*(95% CI)	*β*	*P*	*B*(95% CI)	*β*	*P*	*B*(95% CI)	*β*	*P*	*B*(95% CI)	*β*	*P*
Education, years							6.11 (1.04, 11.18)	0.20	0.019			
Income/person/month, Yuan	7.00 (2.75, 11.25)	0.28	0.001	4.10 (0.39, 7.82)								
Employment status	5.30 (0.17, 10.42)	0.17	0.043				4.58 (0.83, 8.33)	0.20	0.017			
Disease duration, years	−0.46 (−0.82, −0.10)	−0.21	0.012	−0.76 (−1.08, − 0.45)	−0.39	0.000	−0.56 (− 0.82, − 0.29)	−0.35	0.000			
VAS pain	−0.23 (− 0.34, − 0.11)	−0.33	0.000	−0.18 (− 0.29, − 0.08)	−0.29	0.001	−0.17 (− 0.26, − 0.09)	−0.33	0.000			
DAS28										−2.02 (−2.97, −1.08)	−0.39	0.000
Corticosteroids usage				−7.31(−12.79, − 1.84)	−0.21	0.009						
*R* ^2^		0.34			0.40			0.37			0.15	

ǂ Mean of right and left sides. *VAS* Visual analog scale, *DAS28* Disease activity score in 28 joints, *B* Regression coefficient, *95% CI* 95% confidence interval of *B*, *β* Standardized regression coefficient*, R*^*2*^ *R*-squared

## Discussion

This study provided evidence that Chinese RA patients were characterized with reduced wrist ROM, higher Sharp score for the wrists, decreased grip/pinch strength, lower PCS and MCS scores, higher HAQ score, and HADS anxiety and depression scores, which was similar to previous studies from other countries [1–3, 5, 8, 14]. SES, RA disease-specific factors, and drug treatments were significantly associated with wrist ROM. In addition, the ROM measurements might reflect Sharp score for the wrists with regard to the influence factors and negative outcomes. To our knowledge, this is the first study exploring the relationships among SES, disease activity, Sharp score, anxiety/depression, quality of life, and wrist ROM in RA patients.

Khadr Z et al. reported that reduced ROM was associated with old age and female gender in a population of elderly people [7]. In contrast, our study reported that there were no relationships between old age, female gender and lower wrist ROM. One possible explanation for the different results is the existence of cultural diversity and the different participants included in the studies with either Chinese or Western cohorts. Previous studies reported that RA could result in a high economic burden on the individual and the society [47]. Our group reported that SES was significantly associated with patients’ anxiety/depression and quality of life in rheumatic diseases [15, 16]. It was well known that SES is a multifactor. Occupation [48–50], education, and income [51] were frequently used as measures of SES. Whether SES is associated with ROM remains unknown. In the current study, we found that RA patients with lower education level, lower income, lower employment status, and without health insurance were prone to suffer from lower wrist ROM. Due to work-related income reduction, lower education level, and lack of health insurance, RA patients might have a lower adherence rate to medication, which could lead to higher disease activity, more severe joints damage, and loss of physical function [33].

With regard to clinical factors, we found that longer disease duration was significantly associated with lower wrist ROM. This result was in line with the finding of Goodson A and co-workers [8]. When RA progresses, the wrist is increasingly affected [bone erosions and rigid], which possibly lowers the ROM. Furthermore, the current study also revealed that patients with comorbid conditions tended to suffer from reduced wrist ROM, which showed that comorbidity was an important predictor of functional status in RA patients [52]. Pain is a major symptom in RA and is the leading reason for patients seeking medical care [53, 54]. Our study demonstrated significant negative correlations among pain, disease activity, and lower wrist ROM, which were similar to previous study [1]. This result may be attributed to the fact that painful movement and the swelling of soft tissues around the joints are additional important factors contributing to decreased joints mobility in RA. Additionally, we found that there were significant correlations between corticosteroids usage and lower wrist dorsal flexion, which was in contrast with a previous finding [12], which might be explained by the likely dependence of the steroid usage on the more aggressive or refractory forms of RA where corticosteroids were more frequently used. Furthermore, we also found that ESR, CRP, and RF were associated with wrist ROM, which were in line with a previous study [13]. This finding may be attributed to the fact that the higher levels of ESR and CRP, and positive RF result in higher inflammatory activity, causing pain and swelling of the joints.

Interestingly, we found that there were significant association between ROM and Sharp score for the wrists. This finding indicated that ROM measurements might reflect Sharp score for the wrists. However, no causal conclusion could be inferred because the study was cross-sectional in design. Additional clinical trials are required, and the present study just provided a first step towards more focused studies in the future.

To identify which variables were most significantly correlated with lower wrist ROM, a stepwise linear regression analysis was used. Only independent variables individually associated with the primary outcome with a *P*-value < 0.05 were entered into a multivariable regression model. We found that SES, RA disease-specific factors, and drug treatments were significantly associated with wrist ROM, which indicated that SES and RA disease-specific factors were independent risk factors of lower wrist ROM. However, steroid usage is likely to be a consequence of the individual clinical profile with more persistent and/or high disease activity.

However, this study has some limitations. First, the sample size was relatively small and all participants were from a single hospital. Second, the intra- and inter-observer reliabilities of the ROM measurements were not tested. Therefore, it might lead to possible biases of the measurements. However, to minimize the bias, all measurements were taken by two trained physiotherapists under the supervision of a rheumatologist. Third, the inter-rater reliability of Sharp score also could not be tested. However, to ensure the accuracy of Sharp score for the wrists, three rheumatologists evaluated wrist joint damage according to the HSS at the same time, and all readers were blind to the results. In addition, the questionnaires used in this study were all self-reported, which might result in possible biases of the outcomes.

## Conclusions

SES, RA disease-specific factors, and drug treatments were significantly associated with the wrist ROM in RA patients. Additionally, our study suggested that ROM measurements might reflect Sharp score for the wrists with regard to influence factors and outcome measurements. The results indicated that rheumatologists and nurses should be aware of the RA patients’ wrist ROM measurements, especially those with low SES, long disease duration, severe pain, and high disease activity to develop strategies to improve RA patients’ quality of life.

## Abbreviations

ACR: American College of Rheumatology
BMI: Body mass index
BP: Body pain
CI: Confidence intervals
CRP: C-reactive protein
DAS28: Disease activity score in 28 joints
DMARDs: Disease-modifying antirheumatic drugs
ESR: Erythrocyte sedimentation rate
GH: General health
HADS: Hospital Anxiety and Depression Scale
HAQ: Health Assessment Questionnaire
JSN: Joint space narrowing
MCS: Mental Component Summary
MH: Mental health
PCS: Physical Component Summary
PF: Physical function
RA: Rheumatoid arthritis
RE: Role emotional
RF: Rheumatoid factor
ROM: Range of motion
RP: Role physical
SD: Standard deviation
SES: Socioeconomic status
SF: Social function
SF-36: The Short Form 36 Health survey
VAS: Visual analog scale
VT: Vitality
